# Prevalence of Musculoskeletal Disorders among Dentists and Dental Students in Germany

**DOI:** 10.3390/ijerph17238740

**Published:** 2020-11-24

**Authors:** Daniela Ohlendorf, Antonia Naser, Yvonne Haas, Jasmin Haenel, Laura Fraeulin, Fabian Holzgreve, Christina Erbe, Werner Betz, Eileen M. Wanke, Doerthe Brueggmann, Albert Nienhaus, David A. Groneberg

**Affiliations:** 1 Institute of Occupational Medicine, Social Medicine and Environmental Medicine, Goethe-University, Theodor-Stern-Kai 7, 60590 Frankfurt am Main, Germany; Ohlendorf@med.uni-frankfurt.de (D.O.); antonia_naser@web.de (A.N.); yvonne_haas@gmx.de (Y.H.); j.lampe@med.uni-frankfurt.de (J.H.); maltry@med.uni-frankfurt.de (L.F.); wanke@med.uni-frankfurt.de (E.M.W.); brueggmann@med.uni-frankfurt.de (D.B.); groneberg@med.uni-frankfurt.de (D.A.G.); 2 Department of Orthodontics, University Medical Center of the Johannes Gutenberg University, 55131 Mainz, Germany; erbe@uni-mainz.de; 3 Institute of Dentistry, Goethe-University, Theodor-Stern-Kai 7, 60590 Frankfurt am Main, Germany; w.betz@em.uni-frankfurt.de; 4 Competence Center for Epidemiology and Health Services Research for Healthcare Professionals (CVcare), University Medical Center Hamburg-Eppendorf (UKE), Principles of Prevention and Rehabilitation Department (GPR), Institute for Statutory Accident Insurance and Prevention in the Health and Welfare Services (BGW), 20246 Hamburg, Germany; albert.nienhaus@bgw-online.de

**Keywords:** MSD, musculoskeletal pain, prevalence, dentists, dental profession, dental education, Nordic questionnaire

## Abstract

Background: Dentists are at a higher risk of suffering from musculoskeletal disorders (MSD) than the general population. However, the latest study investigating MSD in the dental profession in Germany was published about 20 years ago. Therefore, the aim of this study was to reveal the current prevalence of MSD in dentists and dental students in Germany. Methods: The final study size contained 450 (287 f/163 m) subjects of different areas of specialization. The age of the participants ranged from 23 to 75 years. The questionnaire consisted of a modified version of the Nordic Questionnaire, work-related questions from the latest questionnaire of German dentists, typical medical conditions and self-developed questions. Results: The overall prevalence showed that dentists suffered frequently from MSD (seven days: 65.6%, twelve months: 92%, lifetime: 95.8%). The most affected body regions included the neck (42.7%–70.9%–78.4%), shoulders (29.8%–55.6%–66.2%) and lower back (22.9%–45.8%–58.7%). Overall, female participants stated that they suffered from pain significantly more frequently, especially in the neck, shoulders and upper back. Conclusion: The prevalence of MSD among dentists, especially in the neck, shoulder and back area, was significantly higher than in the general population. In addition, women suffered more frequently from MSD than men in almost all body regions.

## 1. Background

A large body of literature documents the higher risk of dental professionals suffering from musculoskeletal disorders (MSD) or associated pain compared to the general population [1,2,3,4,5,6,7,8,9,10,11,12,13,14,15,16,17,18,19]. The oral cavity represents a very small work area, which is challenging to access and navigate while providing dental care. Thus, dental examinations or treatments require awkward body postures, straining the musculoskeletal system. These positions include flexion of the head and neck to the front and side combined with an inclination and rotation of the trunk towards the patient [20,21,22]. Numerous dental procedures such as the filling of a cavity or the preparation of a root canal require static body postures [23], which are defined as body positions maintained for more than four seconds [24]. As Ohlendorf et al. [21] specified, static positions of the dentist’s head and trunk are generally remained for 27.4% and 23.6% of the treatment time, respectively.

Static muscle forces are found to be more harmful to the musculoskeletal system compared to dynamic forces [25]. In addition, the work of a dentist consists of precise, small and repetitive movements with the hands, often performed with instruments or a vibrating drill. Alexopoulos et al. [2] reported that dentists perceived the high exposure to vibrating tools, repetitive shoulder/hand movements and awkward postures as significant risk factors of their occupation. Due to the exposure to these various physical strains (e.g., awkward positions, static posture, repetitive motions and vibrations) several hours a day and over many years, the susceptibility for MSDs is increased for dental professionals [25,26,27,28,29]. 

One of the earliest studies by Biller in 1946 [30] found that 65% of dentists suffer from back pain. Even today, this particularly high prevalence of musculoskeletal pain has not changed in this occupation. Lietz et al. [19] performed a meta-analysis of 30 studies published between 2005 and 2017 to examine the prevalence of musculoskeletal disease and pain among dental professionals (dentists, dental hygienists and dental students) in Western countries. The prevalence rates of MSDs and pain vary from 10.8% to 97.9%. The authors found that the neck is the body region affected most, since 58.5% of dentists complained about neck pain in the last 12 months. The lower back represented the second most affected body region reported by 56.4% of dentists, followed by shoulder pain (43.1%) and pain in the upper back (41.1%). Among German dentists, Meyer et al. [18] documented that 86.7% of the participants complained of neck and back pain, while 68.6% suffered from musculoskeletal pain during the last 7 days. Worldwide, the findings are all very similar, as they report a high prevalence of MSD and musculoskeletal pain in the dental profession [1,2,3,4,5,6,7,8,9,10,11,12,13,14,15,16,17,18,19].

Historically, the dental profession has been dominated by men [31]. Nowadays, the ratio of female to male dentists is fairly equal (45.6% female versus 54.4% male dentists) [32]. However, regarding the rising numbers of female dental students, a change in demographics is beginning to emerge. In Germany, the number of female students is almost twice as high as the number of male students—with an increasing tendency [33]. This, in fact, makes it crucial to pay particular attention to the needs of female dentists. Unruh’s review of gender variations in clinical pain experience [34] showed that women in general were more likely to experience a variety of recurrent pains. Particularly, female study participants reported more severe levels of pain, more frequent pain and pain of longer duration than men [34]. 

In line with these observations, various researchers found significantly higher levels of musculoskeletal pain among female dentists compared to their male counterparts [1,3,7,35,36]. Valachi et al. [37] identified female dentists as being at an overall higher risk of developing MSD, which was attributed to gender-specific physiological and physical differences. However, inconsistent data exist concerning the affected body parts: while a higher percentage of female dentists suffer from hand and hip pain than males [4], Algahdir et al. [1] observed roughly the same pain prevalence in the lower back, neck and shoulder among female and male dentists. However, female participants reported additional pain in their hands and knees. Moreover, Feng et al. [3] described a significantly increased prevalence of shoulder pain in female dentists. 

Currently, data revealing the MSD prevalence among dentists and dental students in Germany are lacking. In particular, studies focused on gender differences in musculoskeletal pain perception and location are needed. Hence, it is the first aim of this study to provide an update on the MSD prevalence among dentists in Germany. The second aim of the investigation is to focus more on female practitioners and to test whether there are differences regarding pain prevalence or affected body parts in relation to gender.

## 2. Material and Methods

### 2.1. Questionnaire

The questionnaire consists of a modified version of the Nordic questionnaire [38,39,40], questions published by Meyer et al. [18] and self-developed questions. Questions from the Nordic questionnaire assessed musculoskeletal disorder-associated pain of diverse body parts (neck, shoulder, elbow, wrist, upper and lower back, hip, knee and ankle) and their occurrence in the last seven days, last twelve months and over the respondent’s lifetime.

Some questions were extracted from Meyer et al. [18], a survey that was especially developed for the dental occupation. The adopted items ask for the characteristics of the respondent’s working environment and the physical pain they experience that is directly associated with dental work. Moreover, the survey inquiries about pre-existing medical conditions such as rheumatism and arthrosis, carpal and cubital tunnel syndromes, cervical, thoracic and lumbar spine syndromes, tendovaginitis, flexor tendovaginitis and disc prolapse. These conditions are more prevalent in dentists since they are associated with repeated and prolonged musculoskeletal strain while providing dental care [41,42,43,44,45]. In addition, the time of occurrence of the specific medical condition in relation to the practice of dental work was queried. The questionnaire passed through a validation phase. After several circles of revision, the questionnaire was pretested (*n* = 13) successfully.

Exemplary questions are listed in Table 1.

### 2.2. Recruitment

In order to reach a high number of currently practicing dentists, the survey was distributed online via the platform “SoSciSurvey” [46] from May 2018 to May 2019. The Dental Associations of all German federal states were approached and asked to support the survey’s distribution. Furthermore, the survey was promoted via flyers at the “Deutscher Zahnärztetag” (the annual meeting of all German dental societies in Frankfurt am Main, Germany, in 2018) and the “Internationale Dental-Schau” (Dental healthcare fair in in Cologne, Germany, in 2019). Additionally, articles were published to promote the survey in the two dental journals mailed to every practicing dentist in Germany: *Die Zahnarzt Woche* (*DZW*) and *Zahnärztliche Mitteilung* (*ZM*). Due to the support of various dental university departments, it was possible to invite dentists and dental students at universities to participate in the survey. Moreover, the survey was distributed via social media groups that particularly target dental students and junior dentists. 

### 2.3. Subjects

A total of 2548 persons followed the link to the survey and 462 German dentists and dental students completed the questionnaire. Four dental technicians, a dentist who was on parental leave, a student who had already worked as a dental hygienist and two participants who did not report their gender were removed from the analysis. Additionally, the surveys of four undergraduate students were excluded because students do not perform dental treatments in that stage of their education. 

**Figure 1:**
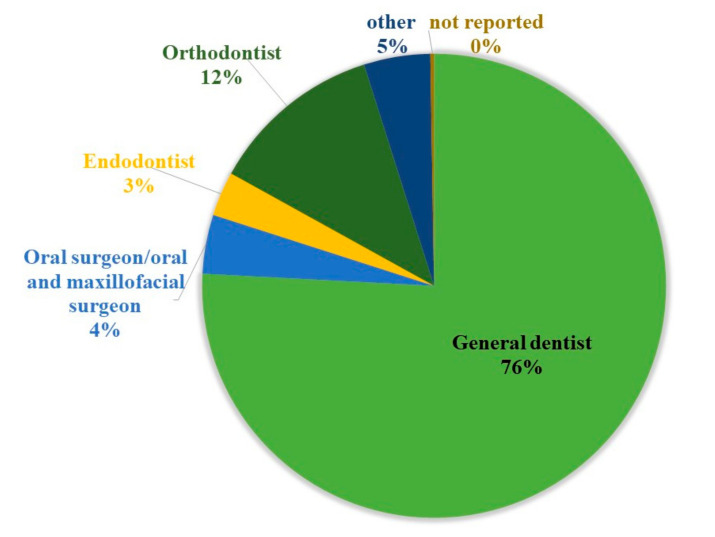
Distribution of dental specialisms from the participating dentists (*n* = 389).

The final sample size contained 450 (287 f/163 m) subjects who filled out the complete questionnaire. The age of the participants ranged from 23 years to 75 years. The distribution between the genders in association with age was representative of the distribution of female versus male dentists in Germany in general, according to which more younger dentists tend to be female [47]. The median age was 35 years (*I*_50_ = 22 years), the median height was 172 cm (*I*_50_ = 13 cm) and the median weight was 68 kg (*I*_50_ = 23 kg) for dentists and students. Among the participants were 61 postgraduate students, 194 dentists who own or share ownership of a practice, 117 dentists who are employed, 55 assistant dentists, 22 dentists at a university hospital and only one dentist working at a hospital. As Figure 1 shows, the sample of dentists (*n* = 389) consisted of 75.8% dental generalists, 12.1% orthodontists, 4.1% oral–maxillofacial surgeons, 3.1% endodontists and 4.6% other specialists such as prosthetic dentists, pedodontists and periodontists (Figure 1). The most common type of office practice most study participants associated with was “single practitioner” office practice (46.3%) followed by a “multi-practitioner” office practice setting (38%), meaning the dentists form an economic and organizational entity. In total, 10.3% of study samples worked in a “shared ownership” practice, meaning the dentists do not form an economic entity, but share the same office space.

The study was approved by the ethics committee of the medical faculty (Goethe-University Frankfurt; No. 356/17). All experiments were performed in accordance with relevant guidelines and regulations.

### 2.4. Statistical Evaluation

The data were extracted from the online surveys and collected in an Excel database [48]. Not plausible answers were removed from the data set. The answer pattern was checked to be logical and plausible, i.e., if pain in the left body part and the right body part were described, the answer was changed to “both body parts”. If pain is stated in the last seven days, it also occurred the “last twelve months” and in “lifetime”. Moreover, when the pain occurred in the “last twelve months”, it indicated its occurrence “in lifetime”. Therefore, a syntax following this comprehensible logic was applied.

The data were analyzed using IMB SPSS Statistics 26 [49]. The metric variables were tested for normal distribution by Kolmogorov–Smirnov testing. For abnormally distributed variables and ordinal data, the median and the interquartile range were defined. Nominal variables were described by frequency. All descriptive statistics were executed for the whole sample, as well separately for female and male participants. We tested whether there was a difference in the frequency of MSD in gender. For this purpose, the overall prevalence of MSD over respondents’ lifetime, the last twelve months and the last seven days was tested by chi^2^ testing for women and men. Additionally, the same test was applied for prevalence in each body location. The test was also applied to reveal whether differences in MSD frequency were present between the right and left sides of the body; therefore, the total sample size of female and male dental professionals was used. We also tested whether there was a difference in the occurrence of general diseases by gender. The chi^2^ testing was applied for the total occurrence of general diseases and the occurrence of each disease in women and men. Additionally, the same test was applied for the occurrence before and after the start of dental practice for the total sample. Fisher’s exact test was applied when 20% of the cells of a contingency table were below 5 participants. Spearman’s correlation was calculated between age and professional experience in years (excluding students), between age and number of MSDs and age and number of general disorders. The significance level was set at 5% and Bonferroni–Holm correction was applied.

## 3. Results

### 3.1. Demographic Variables

Table 2 shows the demographic and work-related characteristics. The age varied between gender: the median age of females was 33 years (*I*_50_ = 17 years) and for males it was 48 years (*I*_50_ = 25.3 years). The median height for female participants was 168 cm (*I*_50_ = 8 cm) and for male participants 182 cm (*I*_50_ = 10 cm). The median weight for female dentists and students was 61 kg (*I*_50_ = 14 kg), whereas it was 83 kg (*I*_50_ = 15.8 kg) for male dentists and students. For female participants, we found a median Body-Mass-Index (BMI) of 21.3 (*I*_50_ = 3.8), which is a relatively low score in the range of a normal BMI defined by the WHO [50]. However, the median BMI for men was 24.7 (*I*_50_ = 4.2), which is a high score in the range of a normal BMI [50]. 

The work-related characteristics of the total sample are listed separately for women and men in Table 3. There was a fairly higher ratio of male dentists who own or share ownership of an office practice (64.4%) compared to female dentists (31%). In accordance, female dentists were more often employed (32.8%) than male dentists (14.1%). However, the ratio of female and male dentists who work in a single-dentist practice was relatively similar (f = 44.2%/m = 49.7%). Male dentists worked more often in a shared practice, which represented no economic entity (f =9.6%/m = 11.4%). In contrast, female dentists worked mostly in a multi-practitioner practice setting that formed an economic entity (f = 40.4%/m = 34.2%). Professional specializations were equally distributed among female and male dentists (Table 2). Male dentists worked longer hours per week (treatment and office work) with a median of 44 h (*I*_50_ = 12 h), compared to female dentists with a median of 38 h (*I*_50_ = 8 h). Moreover, the median duration of professional experience was higher for male dentists ($\tilde{x}$ = 22 years, IQR = 23 years) compared to female dentists ($\tilde{x}$ = 8 years, *I*_50_ = 16 years).

### 3.2. Prevalence of MSD

**Figure 2:**
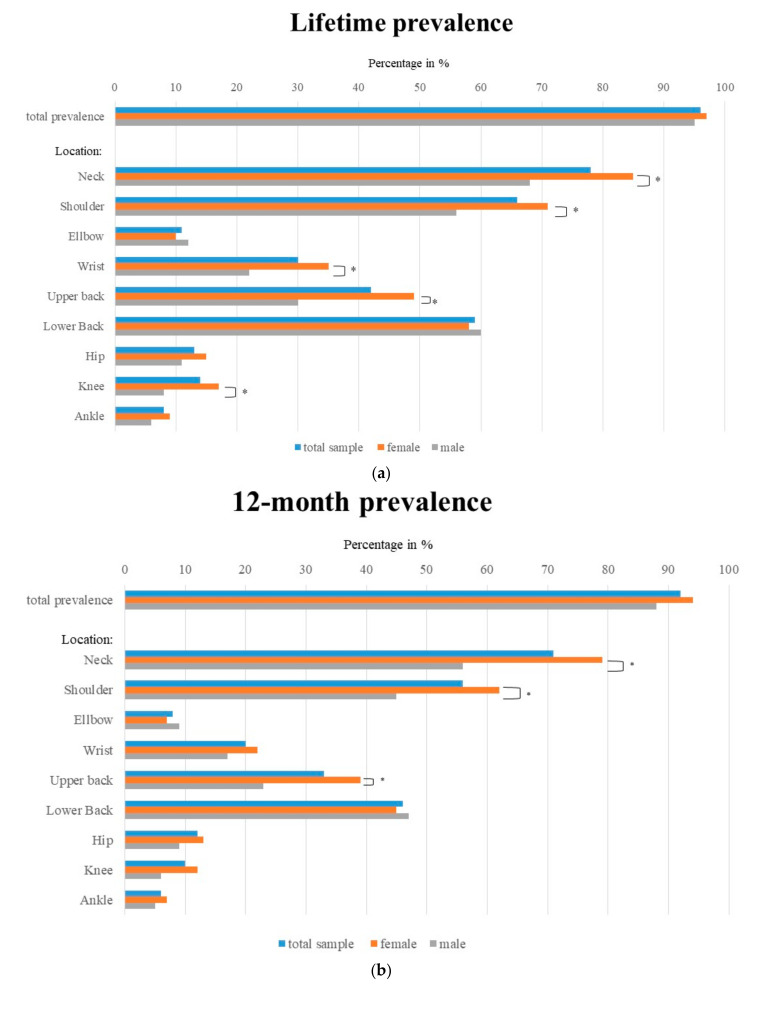

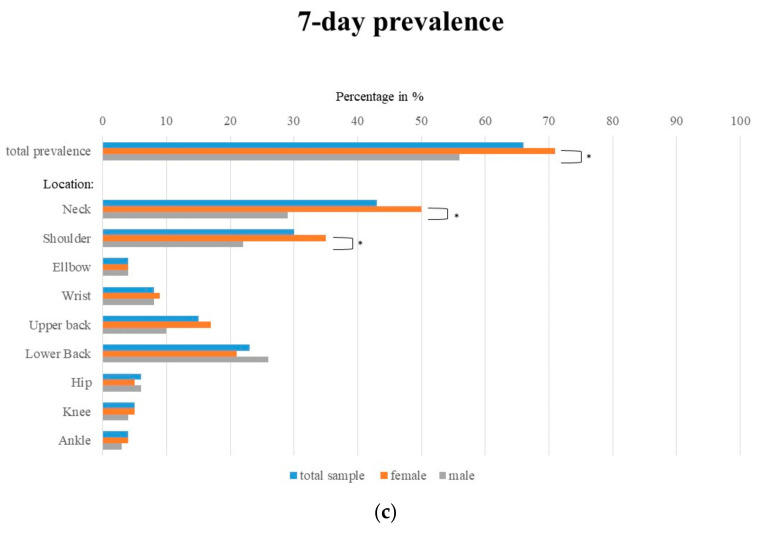
(**a**) Distribution of musculoskeletal disorder (MSD) among all participants (*n* = 450), female participants (*n* = 287) and male participants over lifetime. (**b**) Distribution of MSD among all participants (*n* = 450), female participants (*n* = 287) and male participants for 12-month prevalence. (**c**) Distribution of MSD among all participants (*n* = 450), female participants (*n* = 287) and male participants for 7-day prevalence. * means *p* < 0.05.

Table 4 and Figure 2a–c show the lifetime, 12-month and 7-day prevalence in terms of body regions for all respondents, separated by gender.

Overall, 95.8% of dental professionals (*n* = 431) suffered from musculoskeletal pain in their lifetime. Moreover, 92% of all participants (*n*= 414) reported some musculoskeletal pain in the last twelve months and 65.6% of dental professionals (*n* = 295) stated that they had musculoskeletal pain in the last seven days (Figure 2c). 

Pain was primarily reported on the right side of the body. There was no significant difference between women and men regarding the total pain prevalence either over their lifetime or in the last twelve months. However, we found a significant difference in reporting “pain in the last seven days” between male and female dentists. The neck was the most affected body region in dentists and dental students. Female dentists and female dental students reported neck pain (*p* = 0.001) significantly more often in all evaluated time periods than male dentists and students. 

The second highest prevalence was found for shoulder pain. In total, 65.2% of dentists and dental students suffered from pain in their shoulder at least once in their life. For all three time periods of interest, both shoulders were mostly affected. However, if only one body side was reported to be painful, the right shoulder was mentioned significantly more often (lifetime prevalence *p* = 0.001, 12-month prevalence *p* = 0.05). There were highly significant differences between the two genders in lifetime (*p* = 0.001), 12-month (*p* = 0.01) and 7-day (*p* = 0.01) shoulder pain prevalence. 

The third most common MSD among dentists and dental students affected the lower back. In total, 58.7% of the participants had pain in their lower back at least once in their life. Pain in the lower back did not differ by gender. A slightly lower prevalence was reported in the upper back compared to the lower back. Here, we identified a significant difference between genders. For the time periods “lifetime” and “in the last twelve months”, more female participants suffered pain in their upper back compared to male participants (*p* = 0.001). 

About 30% of all participants reported an experience of wrist pain at least once in their life. Pain in the right wrist was more frequent than pain in the left wrist or both wrists. We found no significant difference in reported pain occurrence between the left and right side. Regarding lifetime prevalence, more female respondents suffered from wrist pain than their male counterparts (*p* = 0.01). 

The lowest prevalence was reported for elbow pain compared with the other inquired anatomical locations of the upper extremities (e.g., lifetime prevalence of 10.7%). Elbow pain was located mostly in the right elbow. However, we found no significant differences for a specific body side or for gender. 

Overall, participants suffered from “pain in the lower body regions” less frequently than “in the upper body regions”. Overall, 13.3% of respondents stated that they experienced hip pain at some time in their life. Pain in the right hip was most common, although we found no significant difference between the right and left hip. Moreover, there was no significant difference in terms of gender. The prevalence for hip pain was similar to the prevalence of knee pain: 13.6% of participants had hip pain in their lifetime. In contrast to hip pain, knee pain was more frequently observed on the left or on both sides. Significantly more female than male dentists/dental students reported to have suffered from knee pain in their lifetime (*p* = 0.01). 

The least frequent reported pain was pain in the ankle region (lifetime prevalence: 7.8%). Ankle pain was felt more frequently in the left ankle or in both ankles. Regarding ankle pain prevalence, we identified no significant difference between female and male participants. 

### 3.3. Typical Medical Conditions 

Overall, 32.2% of all participants reported a diagnosis of at least one of the listed medical conditions (Table 5). Here, we found a trend of more male (33.7%) than female (31.7%) respondents reporting a relevant medical condition. Overall, there was no significant difference in the number of medical conditions reported by female versus male dentists. In general, medical conditions occurred more frequently after having started a daily work routine in the dental profession and there was no significant difference in terms of gender. The median and interquartile distances showing the number of years the disease occurred after the start of respondents’ dental careers are shown in Table 5. 

Cervical spine syndrome was reported by 12.6% of all participants and hence represents the most often mentioned condition (*n* = 57). More than half of the participants suffering from cervical spine syndrome stated that the syndrome occurred after they started working in the dental profession (*n* = 31). About 9.8% of participants (*n* = 44) were diagnosed with a lumbar spine syndrome. Again, more than half of the respondents reported that the syndrome occurred after starting their daily routine in dental care (*n* = 29). Respectively, 7.7% of participants reported a diagnosis of disc prolapse (*n* = 35) or tendovaginitis (*n* = 34), respectively. In both cases, more than half of participants stated that the occurrence was after they started working in the dental profession (*n* = 22; *n* = 21). Furthermore, a comparison between each disease before and after starting work does not reveal any significant differences.

### 3.4. Correlations with Age

The Spearman’s correlation coefficient for age and professional experience in years showed a very strong correlation with r = 0.961 (*p* = 0.001); the correlation coefficient for age and number of general diseases was moderate with r = 0.36 (*p* = 0.001) (Table 6). No correlation was found between the number of body areas where MSDs were reportedly present in “lifetime” (r = 0.03, *p* = 0.56), 12-month (r = −0.09, *p* = 0.06) and 7-day periods (r = 0.03, *p* = 0.49) and age (Table 6). 

## 4. Discussion

The purpose of this study was to identify the current prevalence of musculoskeletal disorders among dentists and dental students in Germany. Regarding the increasing number of females pursuing the dental profession [31,32,33,51], it seems important to carve out differences in MSD occurrence between female and male dentists overall as well as viewed by affected body regions [1,3,4,7,35,36]. 

In our data, the reported total prevalence of musculoskeletal pain was very high, increasing from 7-day (65.6%) to 12-month (92%) to lifetime prevalence (95.8%). This pattern was also seen for pain prevalence in the investigated body regions (Figure 2). In the trunk, the MSD prevalence for 7-day, 12-month and lifetime (neck: 42.7%–70.9%–78.4%, shoulder: 29.8%–55.6%–66.2%, lower back pain: 22.9%–45.8%–58.7%) was higher than in the upper and lower extremities (elbow: 3.6%–8%–10.7%, knee, 4.7%–9.6%–13.6%, ankle: 3.6%–6.2%–7.8%), whereby the wrist (8.4%–20%–30%), especially the right wrist, showed the highest values for the upper extremity.

These high prevalence values, especially in the trunk region, agree with the results of Lietz et al. [19], who published a meta-analysis of 41 studies in Western countries. In these respective data, the neck was the most affected body region (58.5%, 95% CI= 46.0–71.0) throughout the last twelve months followed by the lower back (56.4%, 95% CI = 46.1–66.8), shoulder (43.1%, 95% CI = 30.7–55.5) and upper back (41.1%, 95% CI = 32.3–49.9). A study that investigated MSDs among German dentists in 2001 [18] described the complaint “neck or pain during the last 7 days” in 68.6% of German practitioners. Even more—86.7% of German practitioners—suffered from neck pain or back pain in the last twelve months. It is difficult to compare Meyer et al.’s [18] results to the presented data. The authors did not differentiate between neck and back pain since these two symptoms were evaluated in one question. Our study, however, asked about neck pain, upper back pain and lower back pain separately. For a valid comparison, we summed up the numbers of participants complaining about pain in at least one of the three body parts (neck, upper and lower back). In our study, 56.7% of dentists and dental students reported “neck and back pain in the last seven days” and about 85.6% stated “neck and back pain in the last twelve months”. Although the prevalence of “pain in the last seven days” in our study was about 12% lower than the data published by Meyer et al. [18], the present findings were, overall, very similar to the data collected 20 years ago. 

As a second aim, we investigated the MSD distribution in female and male practitioners since it is commonly known that the perception of pain differs between men and women [34]. While dental practice was dominated by men in the past, in today’s Germany more and more women are working as dentists [33]. Accordingly, the number of female students is almost twice as high as the number of male students with an increasing tendency. In 2018, more women (full-time and part-time) were working in the German health sector than men [52]. In our study, this trend was also reflected in the gender distribution of the respondents, with almost 2/3 women and 1/3 men participating in this survey. Furthermore, the data showed that the participating women were younger and had a lower BMI. The distribution of left and right-handed people between the genders in our study population reflected the general population, meaning that more right-handed persons exist than left-handed. In addition, female dentists stated to have less work experience, fewer total working hours and hours of actual dental treatments per week than men—with proportionally equal time for office work. Furthermore, they were more likely to be employees in an office practice (32.8%). Only 31% stated that they were practice owners compared to 64.4% of their male counterparts, although both genders nevertheless worked most often in a single-dentist practice. This trend can also be seen across the entire German population, with women less likely to be self-employed and more likely to work part-time than men do [52,53]. With regard to the variable total weekly “working time”, “treatment time” and “administration time”, the available data for the work routine of male participants corresponded to the information provided by Meyer et al. [18] almost 20 years ago, although our study found no association between MSD prevalence and practice organization.

The regions of highest MSD prevalence (neck, shoulder, upper and lower back, Table 3, Figure 2) were the same in both sexes. However, the actual prevalence, from a descriptive point of view, was higher in women than in men, even though women work fewer hours per week than men. This is evident for all three time intervals (7-day, 12-month and lifetime) investigated.

Despite the fact that the same patterns for MSDs in individual body regions (the trunk, the right side of the upper extremity and left side lower extremity) existed in men and women, significantly more women than men suffered from pain in the areas of highest prevalence (Table 4; Figure 2a–c). The reasons why the lower back region was the only region where the pain prevalence was minimally higher in men than in women should be investigated in further analyses. However, other studies came to the same conclusion that there were significant differences in MSD occurrence between men and women in the general population and specifically in the dental field [37,54,55]. Explanations for the increased rate of MSDs in females might include a more sensitive perception of pain as well as the influence of sociocultural, psychological and biological factors [56,57,58]. A study from the Netherlands found that hormonal and reproductive factors and estrogen levels are associated with chronic musculoskeletal pain, which makes women more prone to MSD than men [59]. Additionally, the female musculoskeletal system can exert only two-thirds the force than the male system can [60]; therefore, it is more difficult for women to stabilize their body and compensate for the physical strain linked to providing dental care. 

In general, in regard to our data, women report more frequent pain. This is particularly evident in our study by the fact that the symptom “pain in the last 7 days”, regarding all tested regions of the musculoskeletal system, was perceived by more women than men. However, we found no significant gender difference in the number of reported underlying general diseases.

Since the participants were predominantly right-handed (94%), a comparison of the right and left body regions was also carried out for all body regions. The shoulder was the only body region in which a significant difference (lifetime + 12-month time periods) between body sides occurred. The right side was more often affected than the left side. From a descriptive point of view, a uniform pattern of complaints (independent of gender) was identified for all three investigated time periods. We observed a higher pain prevalence on the right side of the body in the shoulder, elbow, wrist and hip and on the contralateral side in the lower extremities (knee and ankle). This pattern is consistent with the posture of dentists during treatment, which has been analyzed in other studies [21,24]. In this context, however, information is missing as to whether the left-handed participants actually work in a left-handed manner, i.e., whether there was a “mirror-inverted” arrangement of the treatment unit, leading to mirror-inverted loading. This aspect should be considered more closely in future analyses. Furthermore, we also consider asking about the more dominant or non-dominant working hand in this context.

Since approximately 94% of the respondents were right-handed in our study population, it can be assumed that the right hand is the hand used to perform demanding and fine motor tasks. Therefore, it is understandable that “pain in the right wrist” was the most commonly reported complaint. However, the work of the left hand should not be disregarded, as it is used for static activities and sometimes also activities involving a certain amount of force, such as holding the cheek or tongue or simply holding a mirror. Due to the double-sided strain, the discrepancy in symptoms between the right and left may be too small to translate into a visible difference. The special role of the wrist is also underlined by the fact that severe pain or restrictions in this region would interfere with or limit the optimal performance of necessary activities significantly. In this context, the question arises as to what therapy could be offered to alleviate musculoskeletal pain and whether the intake of Nonsteroidal anti-inflammatory drugs (NSAID) is useful. However, this question should be investigated in further analyses. 

Dentists are a profession with an exceptionally high prevalence of MSDs, as shown in comparison with a data set collected in the German population [61]. In the latter, the general 12-month prevalence of back pain was 25% in women and 16.9% in men, which was between 20–43% lower than in the present study. When employees in various professions in Germany were studied, a higher percentage—48%—compared to the general population stated that they suffered from pain in the back and neck [62]. This percentage was comparable to the 12-month prevalence of neck and back pain in this study’s sample, but less than our identified lifetime prevalence. In this context, we want to stress that the overall higher prevalence of MSDs in dentists is associated with the numerous MSD risk factors of dental work, which include uninterrupted work, psychologically stressful work, a high number of treated patients, positive correlation with certain dental working tasks, administrative work at a desk, vibrations during work and holding a position for a long time [19]. However, we found a concordance regarding the most painful areas reported between dentists in our sample and the general population (lower back followed by the neck, the shoulder and the knee) [63]. 

An important question arises as to whether the complaints worsen with age or work experience. However, numerous studies report that MSDs increase with age [17]. This could not be confirmed in our data set since no correlation was present between age and the occurrence of MSD in different body areas over any period of time (7-day, 12-month, lifetime). We observed a very strong correlation between age and job experience (*p* = 0.001) in our data; the same can be assumed for the correlation between the number of MSDs in different body areas and job experience. However, studies exist that are in line with our findings and have even shown slightly negative correlations between age/job experience and MSDs [14,15,64]. Furthermore, the observation of a non-correlation is supported by the fact that even dental students suffer from MSD [65,66]. A possible explanation for the lacking effect of age on MSD prevalence could be a better adjusted working position and techniques implemented by experienced dentists to avoid musculoskeletal problems [67]. Furthermore, it is also possible that those dentists suffering from severe MSD retire early and the healthier ones continue working in the profession. However, a moderate correlation between age and the number of typical medical conditions was found since complaints of different body regions accumulate with increasing age.

In total, the age distribution of participants matched the available official demographic data of practicing dentists in Germany who are more likely to be female and younger than their male colleagues [47]. With such a young group of subjects (median age: 39.5 years) it can be assumed that they are also more acquainted to filling out online questionnaires than older people. Whether the same findings would have resulted in a paper–pencil version of the survey or whether elderly practitioners would have taken part in the online version at all remains unclear. However, since age is not correlated with MSD prevalence, no change in MSD prevalence would be expected in a potential older study population. Since about 2/3 of the participants were women, this may also be related to the high MSD prevalence in our sample. In this context, it should also be taken into account that there is a different perception of pain between men and women and therefore female participants might have a greater interest in this topic. However, since this distribution of participants reflects the reality in Germany, a certain external validity of the gathered data can be assumed. 

Future research is necessary, not only in relation to dental equipment or the ergonomic environment, but also focused on those dentists who suffer from MSD versus the ones who do not. The many differences in MDS occurrence might be due to different working postures, setups or habits (using indirect working or surgical loupes, adjusting the patient and the dentist’s position optimally or taking breaks regularly), a divergent arrangement of the work inventory, the training condition of the musculoskeletal system or cooperation with the dental assistant. Therefore, personal and professional characteristics must be considered more closely. Protective behavior could include attending ergonomic training, relaxing training or learning about coping strategies to reduce stress at work and after work. After identifying possible preventive and protective factors, these must be tested in a follow-up study to prove these effects. 

Above all, our knowledge in relation to ergonomic working has constantly increased [68,69]. However, a great need for improvement remains in relation to dental professionals implementing ergonomic habits more effectively in their daily working routine, especially because these habits affect their own health directly. The results of the present study confirm the findings of many international studies [1,2,3,4,5,6,7,8,9,10,11,12,13,14,15,16,17,18,19], which report a worldwide high prevalence of MSDs and pain in the dental profession. These rates of MSD prevalence are especially high in female dentists and have remained the same for the past 20 years [18].

## 5. Conclusions

In German dentists and dental students, the prevalence of MSDs—especially in the neck, shoulder and back area—was found to be very high and higher than the observed rates in the German population. Female dental professionals reported an even higher MSD prevalence than their male counterparts regarding all body regions surveyed. Furthermore, female dentists were younger, had a lower BMI and worked, in general, fewer hours per week. Consequently, such factors should be taken into account when behavioral preventive measures, e.g., the design of job-related strength training, or the therapy of MSDs is implemented.

## Figures and Tables

**Table 1:** Exemplary questions from the questionnaire.

Questions	Possible Answers
What is your field of specialization?Please select the field of specialization in which you are mainly working.	General dentist
	Oral surgeon/oral and maxillofacial surgeon
	Endodontist
	Orthodontist
	Other:
Which occupational group do you belong to:	Owner or shared ownership of a practice and Practicing dentist
	Assistant dentist
	Employed dentist
	Dentist at a university hospital
	Dentist at a hospital
	Student of the clinical section
	Other:
How many hours a week do you work on average at your workplace?	
- Total working hours	approx.__ hours per week
- Dental treatment time	approx.__ hours per week
- Administrative work (office work)	approx.__ hours per week
Have you had any discomfort or pain in the following areas of your body at any time? Please select the appropriate body regions.	Neck region
	Shoulder region
	Elbow region
	Wrists/Hands
	Upper back/thoracic spine
	Lower back (lumbar spine)
	Hips/Thighs
	Knee
	Ankles/Feet
	No, there were no complaints or pain.

**Table 2:** Demographic characteristics of all participants with a total sample size of *n* = 450.

Demographic Characteristics	Total Sample	Female	Male
**Sex**			
*n* (%)	450 (100)	287 (63.8)	163 (36.2)
**Age (Years)**			
$\tilde{x}$ ($I_{50}$)	35 (22)	33 (17)	48 (25)
min	19	20	19
max.	75	65	75
**Height (cm)**			
$\tilde{x}$ ($I_{50}$)	172 (13)	168 (8)	182 (10)
**Weight (kg)**			
$\tilde{x}$ ($I_{50}$)	68 (23)	61(14)	83 (15.8)
**BMI (kg/m^2^)**			
$\tilde{x}$ ($I_{50}$)	22.7 (4.9)	21.3 (3.8)	24.7 (4.2)
**Handedness**			
*n* (%)	450 (100)	287 (100)	163 (100)
right	423 (94)	271 (94.4)	152 (93.8)
left	26 (5.8)	16 (5.6)	10 (6.1)
not reported	1 (0.2)	0 (0)	1 (0.6)

**Table 3:** Work-related characteristics of the total sample and comparison between female and male participants.

Work-Related Characteristics	Total Sample	Female	Male
***n*** **(%)**	450 (100)	287 (100)	163 (100)
**Professional group**			
Owner or shared ownership of a practice and practicing dentist	194 (43.1)	89 (31)	105 (64.4)
Assistant dentist	55 (12.2)	40 (13.9)	15 (9.2)
Employed dentist	117 (26)	94 (32.8)	23 (14.1)
Dentist at a university hospital	22 (4.9)	17 (5.9)	5 (3.1)
Dentist at a hospital	1 (0.2)	0 (0)	1 (0.6)
Student of the clinical section	61 (13.6)	47 (16.4)	14 (8.6)
**weekly absolute time of work (h)**			
$\tilde{x}$ ($I_{50}$)	40 (10)	38 (8)	44 (12)
not reported	34 (7.5)	22 (7.7)	12 (7.4)
**weekly time of dental treatment (h)**			
$\bar{x}$ ($I_{50})$	32 (10)	30 (15)	35 (7)
not reported	32 (7.1)	20 (7)	12 (7.4)
**weekly time of work in office (h)**			
$\tilde{x}$ ($I_{50}$)	6 (6)	5 (7)	8 (7)
not reported	37 (8.2)	24 (8.4)	13 (8)
**Only approbated dentists**			
*n* (%)	389 (100)	240 (100)	149 (100)
**Practice type in *n* (%)**			
Solo dentistry practice	180 (46.3)	106 (44.2)	74 (49.7)
Practice sharing	40 (10.3)	23 (9.6)	17 (11.4)
Multi-practitioner practice	148 (38)	97 (40.4)	51 (34.2)
not reported	21 (5.4)	14 (5.8)	7 (4.7)
**Professional experience in years**			
$\tilde{x}$ ($I_{50}$)	10.5 (21)	8 (16)	22 (23)
**Specialty in *n* (%)**			
General dentist	295 (75.8)	181 (75.4)	114 (76.5)
Oral surgeon/oral and maxillofacial surgeon	16 (4.1)	5 (2.1)	11 (7.4)
Endodontist	12 (3.1)	4 (1.7)	8 (5.4)
Orthodontist	47 (12.1)	34 (14.2)	13 (8.7)
other	18 (4.6)	15 (6.3)	3 (2)
not reported	1 (0.3)	1 (0.4)	0 (0)

**Table 4:** Lifetime, 12-month and 7-day prevalence in terms of body regions for all respondents, separated by gender. An asterisk after the chi^2^ value symbolizes the significance level (* *p* < 0.05, ** *p* < 0.01, *** *p* < 0.001). An ‘^a^’ stands behind the chi^2^ test, if Fisher’s exact test was applied when 20% of the cells of a contingency table were below 5.

		Lifetime Prevalence *n* (%)					12-Month Prevalence *n* (%)					7-day Prevalence *n* (%)				
		Total	Female	Male	Chi^2^ Female/Male	Chi^2^ Right/Left	Total	Female	Male	Chi^2^ Female/Male	Chi^2^ Right/Left	Total	Female	Male	Chi^2^ Female/Male	Chi^2^ Right/Left
**Total prevalence**		431 (95.8)	277 (96.5)	154 (94.5)	0.334		414 (92)	270 (94.1)	144 (88.3)	0.108		295 (65.6)	203 (70.7)	92 (56.4)	0.007 **	
**Neck**		353 (78.4)	243 (84.7)	110 (67.5)	0.001 ***		319 (70.9)	227 (79.1)	92 (56.4)	0.001 ***		192 (42.7)	144 (50.2)	48 (29.4)	0.001 ***	
**Shoulder**	Total	298 (66.2)	206 (70.8)	92 (56.4)	0.001 ***		250 (55.6)	177 (61.7)	73 (44.8)	0.002 **		134 (29.8)	99 (34.5)	35 (21.5)	0.006 **	
	Left	48 (10.7)	28 (9.8)	20 (12.3)	0.429	0.001 ***	51 (11.3)	33 (11.5)	18 (11)	1.000	0.012 *	29 (6.4)	21 (7.3)	8 (4.9)	0.425	0.059
	Right	86 (19.1)	58 (20.2)	28 (17.2)	0.457		82 (18.2)	59 (20.6)	23 (14.1)	0.125		46 (10.2)	35 (12.2)	11 (6.7)	0.103	
	Both	164 (36.4)	120 (41.8)	44 (27)	0.002 **		137 (30.4)	103 (35.9)	34 (20.9)	0.002 **		59 (13.1)	43 (15)	16 (9.8)	0.187	
**Elbow**	Total	48 (10.7)	28 (9.8)	20 (12.3)	0.429		36 (8.0)	21 (7.3)	15 (9.2)	0.468		16 (3.6)	10 (3.5)	6 (3.7)	1.000	
	Left	6 (1.3)	2 (0.7)	4 (2.5)	0.196 ^a^	1.000 ^a^	5 (1.1)	3 (1)	2 (1.2)	0.671 ^a^	1.000 ^a^	2 (0.4)	2 (0.7)	0 (0)	0.541 ^a^	1.000 ^a^
	Right	27 (6.0)	19 (6.6)	8 (4.9)	0.540		20 (4.4)	13 (4.5)	7 (4.3)	1.000		13 (2.9)	7 (2.4)	6 (3.7)	0.557	
	Both	15 (3.3)	7 (2.4)	8 (4.9)	0.178		11 (2.4)	5 (1.7)	6 (3.7)	0.210 ^a^		1 (0.2)	1 (0.3)	0 (0)	1.000 ^a^	
**Wrist**	Total	135 (30)	99 (34.5)	36 (22.1)	0.007 **		90 (20)	62 (21.6)	28 (17.2)	0.326		38 (8.4)	25 (8.7)	13 (8)	0.862	
	Left	16 (3.6)	11 (3.8)	5 (3.1)	0.795	0.086 ^a^	10 (2.2)	8 (2.8)	2 (1.2)	0.506 ^a^	0.619 ^a^	1 (0.2)	1 (0.3)	0 (0)	1.000 ^a^	1.000 ^a^
	Right	75 (16.7)	58 (20.2)	17 (10.4)	0.008 **		53 (11.8)	38 (13.2)	15 (9.2)	0.284		26 (5.8)	17 (5.9)	9 (5.5)	1.000	
	Both	44 (9.8)	30 (10.5)	14 (8.6)	0.621		28 (6.2)	17 (5.9)	11 (6.7)	0.686		11 (2.4)	7 (2.4)	4 (2.5)	1.000 ^a^	
**Upper back**		190 (42.2)	141 (49.1)	49 (30.1)	0.001 ***		150 (33.3)	113 (39.4)	37 (22.7)	0.001 ***		67 (14.9)	50 (17.4)	17 (10.4)	0.071	
**Lower Back**		264 (58.7)	166 (57.8)	98 (60.1)	0.691		206 (45.8)	130 45.3	76 (46.6)	0.615		103 (22.9)	61 (21.3)	42 (25.8)	0.238	
**Hip**	Total	60 (13.3)	42 (14.6)	18 (11)	0.315		52 (11.6)	37 (12.9)	15 (9.2)	0.287		25 (5.6)	15 (5.2)	10 (6.1)	0.669	
	Left	18 (4)	11 (3.8)	7 (4.3)	1.000	0.614 ^a^	17 (3.8)	10 (3.5)	7 (4.3)	0.797	1.000 ^a^	9 (2.0)	5 (1.7)	4 (2.5)	0.727 ^a^	1.000 ^a^
	Right	26 (5.8)	22 (7.7)	4 (2.5)	0.033 *		23 (5.1)	20 (7)	3 (1.8)	0.024 *		12 (2.7)	9 (3.1)	3 (1.8)	0.551 ^a^	
	Both	16 (3.6)	9 (3.1)	7 (4.3)	0.599		12 (2.7)	7 (2.4)	5 (3.1)	0.762 ^a^		4 (0.9)	1 (0.3)	3 (1.8)	0.131 ^a^	
**Knee**	Total	61 (13.6)	48 (16.7)	13 (8)	0.010 **		43 (9.6)	33 (11.5)	10 (6.1)	0.093		21 (4.7)	15 (5.2)	6 (3.7)	0.642	
	Left	19 (4.2)	13 (4.5)	6 (3.7)	0.809	1.000 ^a^	16 (3.5)	12 (4.2)	4 (2.4)	0.621	0.437 ^a^	10 (2.2)	7 (2.4)	3 (1.8)	1.000 ^a^	1.000 ^a^
	Right	16 (3.6)	15 (5.2)	1 (0.6)	0.014 *		13 (2.9)	12 (4.2)	1 (0.6)	0.023 ^a^ *		4 (0.9)	4 (1.4)	0 (0)	0.302 ^a^	
	Both	26 (5.8)	20 (7)	6 (3.7)	0.207		14 (3.1)	9 (3.1)	5 (3.1)	1.000		7 (1.6)	4 (1.4)	3 (1.8)	0.703 ^a^	
**Ankle**	Total	35 (7.8)	26 (9.1)	9 (5.5)	0.203		28 (6.2)	20 (7)	8 (4.9)	0.541		16 (3.6)	11 (3.8)	5 (3.1)	0.796	
	Left	11 (2.4)	8 (2.8)	3 (1.8)	0.753	1.000 ^a^	7 (1.6)	5 (1.7)	2 (1.2)	0.717 ^a^	1.000 ^a^	5 (1.1)	4 (1.4)	1 (0.6)	0.660 ^a^	1.000 ^a^
	Right	6 (1.3)	4 (1.4)	2 (1.2)	1.000		6 (1.3)	3 (1)	3 (1.8)	0.671 ^a^		3 (0.7)	1 (0.3)	2 (1.2)	0.289 ^a^	
	Both	18 (4)	14 (4.9)	4 (2.5)	0.226		15 (3.3)	12 (4.2)	3 (1.8)	0.275		8 (1.8)	6 (2.1)	2 (1.2)	0.717 ^a^	

**Table 5:** General diseases in terms of their occurrence for all respondents, separated by gender. An asterisk after the chi^2^ value symbolizes the significance level (* *p*< 0.05, ** *p* < 0.01, *** *p* < 0.001). An ‘^a^’ stands behind the chi^2^ test, if Fisher’s exact test was applied when 20% of the cells of a contingency table were below 5.

Disease	Occurrence *n* (%)				Occurrence before Start of Profession *n* (%)				Occurrence after Start of Profession *n* (%)				Occurrence after xx Years Min/Max		
	Total	Female	Male	Chi^2^ Female/Male	Total	Female	Male	Chi^2^ Female/Male	Total	Female	Male	Chi^2^ Female/Male	Total	Female	Male
**Total *n* (%)**	146 (32.4)	91 (31.7)	55 (33.7)	0.676											
**Rheumatism**	3 (0.7)	2 (0.7)	1 (0.6)	1.000 ^a^	1 (0.2)	0 (0)	1 (0.6)	0.362 ^a^	2 (0.4)	2 (0.7)	0 (0)	0.537 ^a^	18/20	18/20	
**Arthrosis**	20 (4.4)	10 (3.5)	10 (6.1)	0.234	3 (0.7)	2 (0.7)	1 (0.6)	1.000 ^a^	14 (3.1)	6 (2.1)	8 (4.9)	0.155	5/35	5/20	5/35
**Carpal Tunnel Syndrome**	14 (3.1)	11 (3.8)	3 (1.8)	0.276	3 (0.7)	2 (0.7)	1 (0.6)	1.000 ^a^	9 (2.0)	7 (2.4)	2 (1.2)	0.498 ^a^	2/20	2/20	15/20
**Disc Prolapse**	35 (7.8)	20 (7)	15 (9.2)	0.464	7 (1.6)	6 (2.1)	1 (0.6)	0.430 ^a^	22 (4.9)	10 (3.5)	12 (7.4)	0.073	2/40	2/22	4/40
**Tendovaginitis**	34 (7.6)	21 (7.3)	13 (8)	0.853	10 (2.2)	7 (2.4)	3 (1.8)	1.000 ^a^	21 (4.7)	12 (4.2)	9 (5.5)	0.643	1/30	1/23	5/30
**Flexor Tendovaginitis**	4 (0.9)	3 (1)	1 (0.6)	1.000 ^a^	0(0)	0 (0)	0 (0)	_____	2 (0.4)	1 (0.3)	1 (0.6)	1.000	6/20	6/20	
**Cubital Tunnel Syndrome**	0 (0)	0 (0)	0 (0)	______	0 (0)	0 (0)	0(0)	_____	0 (0)	0(0)	0 (0)	_______	_______	______	______
**Cervical Spine Syndrome**	57 (12.7)	41 (14.3)	16 (9.8)	0.187	15 (3.3)	11 (3.8)	4 (2.5)	0.588	31 (6.9)	20 (7)	11 (6.7)	1.000	1/27	1/27	1/25
**Thoracic Spine Syndrome**	14 (3.1)	11 (3.8)	3 (1.8)	0.276	1 (0,2)	1 (0.3)	0 (0)	1.000 ^a^	10 (2.2)	7 (2.4)	3 (1.8)	1.000 ^a^	1/25	1/25	10/15
**Lumbar Spine Syndrome**	44 (9.8)	27 (9.4)	17 (10.4)	0.743	10 (2.2)	8 (2.8)	2 (1.2)	0.341 ^a^	29 (6.4)	16 (5.6)	13 (8)	0.425	1/401	1/20	3/40

**Table 6:** Prevalence, number of general diseases as well as professional experience (in years) correlated by age.

Prevalence, Number of General Diseases, Professional Experience	Spearman-Rho	Significance *p*
Lifetime prevalence	0.03	0.56
12-month prevalence	−0.09	0.06
7-day prevalence	0.03	0.49
Number of Typical medical conditions	0.36	0.001
Professional experience in years	0.94	0.001

## References

[1] Alghadir A., Zafar H., Iqbal Z.A. (2015). Work-related musculoskeletal disorders among dental professionals in Saudi Arabia. J. Phys. Ther. Sci..

[2] Alexopoulos E.C., Stathi I.C., Charizani F. (2004). Prevalence of musculoskeletal disorders in dentists. BMC Musculoskelet. Disord..

[3] Feng B., Liang Q., Wang Y., Andersen L.L., Szeto G. (2014). Prevalence of work-related musculoskeletal symptoms of the neck and upper extremity among dentists in China. BMJ Open.

[4] Finsen L., Christensen H., Bakke M. (1998). Musculoskeletal disorders among dentists and variation in dental work. Appl. Ergon..

[5] Golchha V., Sharma P., Wadhwa J., Yadav D., Paul R. (2014). Ergonomic risk factors and their association with musculoskeletal disorders among Indian dentist: A preliminary study using Rapid Upper Limb Assessment. Indian J. Dent. Res..

[6] Gopinadh A., Devi K.N., Chiramana S., Manne P., Sampath A., Babu M.S. (2013). Ergonomics and musculoskeletal disorder: As an occupational hazard in dentistry. J. Contemp. Dent. Pract..

[7] Sustova Z., Hodacova L., Kapitan M. (2013). The prevalence of musculoskeletal disorders among dentists in the Czech Republic. Acta Medica (Hradec Kralove).

[8] Hodacova L., Sustova Z., Cermakova E., Kapitan M., Smejkalova J. (2015). Self-reported risk factors related to the most frequent musculoskeletal complaints among Czech dentists. Ind. Health.

[9] Kierklo A., Kobus A., Jaworska M., Botulinski B. (2011). Work-related musculoskeletal disorders among dentists—A questionnaire survey. Ann. Agric. Environ. Med..

[10] Kumar V.K., Kumar S.P., Baliga M.R. (2013). Prevalence of work-related musculoskeletal complaints among dentists in India: A national cross-sectional survey. Indian J. Dent. Res..

[11] Nokhostin M.R., Zafarmand A.H. (2016). “Musculoskeletal problem”: Its prevalence among Iranian dentists. J. Int. Soc. Prev. Community Dent..

[12] Rafie F., Zamani Jam A., Shahravan A., Raoof M., Eskandarizadeh A. (2015). Prevalence of Upper Extremity Musculoskeletal Disorders in Dentists: Symptoms and Risk Factors. J. Environ. Public Health.

[13] Tirgar A., Javanshir K., Talebian A., Amini F., Parhiz A. (2015). Musculoskeletal disorders among a group of Iranian general dental practitioners. J. Back Musculoskelet. Rehabil..

[14] Leggat P.A., Smith D.R. (2006). Musculoskeletal disorders self-reported by dentists in Queensland, Australia. Aust. Dent. J..

[15] Chowanadisai S., Kukiattrakoon B., Yapong B., Kedjarune U., Leggat P.A. (2000). Occupational health problems of dentists in southern Thailand. Int. Dent. J..

[16] Dajpratham P., Ploypetch T., Kiattavorncharoen S., Boonsiriseth K. (2010). Prevalence and associated factors of musculoskeletal pain among the dental personnel in a dental school. J. Med. Assoc. Thai.

[17] Ratzon N.Z., Yaros T., Mizlik A., Kanner T. (2000). Musculoskeletal symptoms among dentists in relation to work posture. Work.

[18] Meyer V.P., Brehler R., Castro W.H.M., Nentwig C.G. (2001). Arbeitsbelastungen bei Zahnärzten in niedergelassener Praxis. Eine arbeitsmedizinische Bestandsaufnahme zu Wirbelsäulenbelastungen, Berufsdermatosen und Stressfaktoren.

[19] Lietz J., Kozak A., Nienhaus A. (2018). Prevalence and occupational risk factors of musculoskeletal diseases and pain among dental professionals in Western countries: A systematic literature review and meta-analysis. PLoS ONE.

[20] De Sio S., Traversini V., Rinaldo F., Colasanti V., Buomprisco G., Perri R., Mormone F., La Torre G., Guerra F. (2018). Ergonomic risk and preventive measures of musculoskeletal disorders in the dentistry environment: An umbrella review. PeerJ.

[21] Ohlendorf D., Erbe C., Nowak J., Hauck I., Hermanns I., Ditchen D., Ellegast R., Groneberg D.A. (2017). Constrained posture in dentistry—A kinematic analysis of dentists. BMC Musculoskelet. Disord..

[22] Akesson I., Hansson G.A., Balogh I., Moritz U., Skerfving S. (1997). Quantifying work load in neck, shoulders and wrists in female dentists. Int. Arch. Occup. Environ. Health.

[23] Nowak J., Erbe C., Hauck I., Groneberg D.A., Hermanns I., Ellegast R., Ditchen D., Ohlendorf D. (2016). Motion analysis in the field of dentistry: A kinematic comparison of dentists and orthodontists. BMJ Open.

[24] Ohlendorf D., Erbe C., Hauck I., Nowak J., Hermanns I., Ditchen D., Ellegast R., Groneberg D.A. (2016). Kinematic analysis of work-related musculoskeletal loading of trunk among dentists in Germany. BMC Musculoskelet. Disord..

[25] Valachi B., Valachi K. (2003). Mechanisms leading to musculoskeletal disorders in dentistry. J. Am. Dent. Assoc..

[26] Bernard B. (1997). Musculoskeletal Disorders and Workplace Factors.

[27] Keyserling W.M. (2000). Workplace risk factors and occupational musculoskeletal disorders, Part 2: A review of biomechanical and psychophysical research on risk factors associated with upper extremity disorders. AIHAJ.

[28] Punnett L., Wegman D.H. (2004). Work-related musculoskeletal disorders: The epidemiologic evidence and the debate. J. Electromyogr. Kinesiol..

[29] Keyserling W.M. (2000). Workplace risk factors and occupational musculoskeletal disorders, Part 1: A review of biomechanical and psychophysical research on risk factors associated with low-back pain. AIHAJ.

[30] Biller F. (1946). Occupational hazards in dental practice. Oral Hyg..

[31] Kuhlmann E. (1999). Profession und Geschlechterdifferenz. Eine Studie über die Zahnmedizin.

[32] Daten & Fakten 2020. https://www.kzbv.de/kzbv-daten-fakten-2020-web.media.03365edd349fafa25f6c84deeb8dc251.pdf.

[33] Statista Anzahl der Studierenden im Fach Zahnmedizin in Deutscland nach Gesclecht in den Wintersemestern von 2007/2008 bis 2018/2019. https://de.statista.com/statistik/daten/studie/200762/umfrage/entwicklung-der-anzahl-der-zahnmedizinstudenten/.

[34] Unruh A.M. (1996). Gender variations in clinical pain experience. Pain.

[35] Kurşun Ş., Evirgen S., Akbulut N., Oztas B., Vaizoglu S.A. (2014). Work Characteristics and Musculoskeletal Disorders among Postgraduate Dental Students: A Pilot Study. J. Musculoskeletal. Pain.

[36] Rundcrantz B.L., Johnsson B., Moritz U. (1991). Occupational cervico-brachial disorders among dentists. Analysis of ergonomics and locomotor functions. Swed. Dent. J..

[37] Valachi B. (2008). Musculoskeletal health of the woman dentist: Distinctive interventions for a growing population. J. Calif. Dent. Assoc..

[38] Kuorinka I., Jonsson B., Kilbom A., Vinterberg H., Biering-Sorensen F., Andersson G., Jorgensen K. (1987). Standardised Nordic questionnaires for the analysis of musculoskeletal symptoms. Appl. Ergon..

[39] Slesina W. (1987). Fragebogen zur Subjektiven Einschätzung der Belastungen am Arbeitsplatz.

[40] Caffier G., Steinberg U., Liebers F. (1999). Praxisorientiertes Methodeninventar zur Belastungs- und Beanspruchungsbeurteilung im Zusammenhang mit Arbeitsbedingten Muskel-Skelett-erkrankungen.

[41] Solovieva S., Vehmas T., Riihimaki H., Takala E.P., Murtomaa H., Luoma K., Leino-Arjas P. (2006). Finger osteoarthritis and differences in dental work tasks. J. Dent. Res..

[42] Reitemeier B., Jatzwauk L., Neumann K., Schneevoigt R., Kleine J., Tzscheutschler C., Körnig T. (2012). Zahnärztliche Handinstrumente- ergonomische und hygienische Aspekte. ZMK. https://www.zmk-aktuell.de/fachgebiete/hygiene/story/zahnaerztliche-handinstrumente--ergonomische-und-hygienische-aspekte__643.html?sword=reitemeier.

[43] Lake J. (1995). Musculoskeletal dysfunction associated with the practice of dentistry--proposed mechanisms and management: Literature review. Univ. Tor. Dent. J..

[44] Valachi B. (2018). Wrist Pain among Dental Professionals.

[45] Adams J.E., Habbu R. (2015). Tendinopathies of the Hand and Wrist. J. Am. Acad Orthop. Surg..

[46] Leitner D.J. (2019). SoSci Survey; Version 3.1.06. https://www.soscisurvey.de.

[47] Bundeszahnärztekammer Altersverteilung. https://www.bzaek.de/ueber-uns/daten-und-zahlen/mitgliederstatistik/altersverteilung/.

[48] Microsoft Corp (2018). Microsoft Excel for Mac, Version 16.18.

[49] IBM Corp (2019). IBM SPSS Statistics for Mac.

[50] WHO Body Mass Index-BMI. https://www.euro.who.int/en/health-topics/disease-prevention/nutrition/a-healthy-lifestyle/body-mass-index-bmi.

[51] Bundeszahnärztekammer (2018). Statistisches Jahrebuch.

[52] Bundesagentur für Arbeit Statistik/Arbeitsmarktberichterstattung (2019). Die Arbeitsmarktsituation von Frauen und Männern 2018.

[53] Kümmerling A., Postels D., Slomka C. (2015). Arbeitszeiten von Männern und Frauen- alles wie gehabt? Analysen zur Erwerbsbeteiligung in Ost- und Westdeutschland.

[54] Lundberg U. (2002). Psychophysiology of work: Stress, gender, endocrine response, and work-related upper extremity disorders. Am. J. Ind. Med..

[55] Proteau R.-A. (2009). Prevention of Work-Related Musculoskeletal Disorders (MSDs) in Dental Clinics.

[56] Wiesenfeld-Hallin Z. (2005). Sex differences in pain perception. Gend. Med..

[57] Dao T.T., LeResche L. (2000). Gender differences in pain. J. Orofac. Pain.

[58] Hallin R.G. (2003). Pain more painful in women. Gender perspective neglected in research on the biological mechanisms of pain. Lakartidningen.

[59] Wijnhoven H.A., de Vet H.C.W., Smit H.A., Picavet H.S.J. (2006). Hormonal and reproductive factors are associated with chronic low back pain and chronic upper extremity pain in women—The MORGEN study. Spine.

[60] Grandjean E., Kroemer K.H.E. (1997). Fitting The Task to the Human: A Textbook of Occupational Ergonomics.

[61] RKI (2015). Gesundheitsberichtersteattung des Bundes Gemeinsam Getragen von RKI und Destatis—Gesundheit in Deutschland.

[62] Steinke M., Badura B. (2011). Präsentismus: Das kann teuer Werden. Die BKK 04/2011, 247-251.

[63] Liebscher-Bracht R., Liebscher P. Verteilung von Schmerz in Deutschland nach Körperregion und Geschlecht im Jahr 2017. https://de.statista.com/statistik/daten/studie/896807/umfrage/verteilung-von-schmerz-in-deutschland-nach-koerperregion-und-geschlecht/.

[64] Burdorf A., Sorock G. (1997). Positive and negative evidence of risk factors for back disorders. Scand. J. Work Environ. Health.

[65] Thornton L.J., Barr A.E., Stuart-Buttle C., Gaughan J.P., Wilson E.R., Jackson A.D., Wyszynski T.C., Smarkola C. (2008). Perceived musculoskeletal symptoms among dental students in the clinic work environment. Ergonomics.

[66] Rising D.W., Bennett B.C., Hursh K., Plesh O. (2005). Reports of body pain in a dental student population. J. Am. Dent. Assoc..

[67] Leggat P.A., Kedjarune U., Smith D.R. (2007). Occupational health problems in modern dentistry: A review. Ind. Health.

[68] Valachi B., Valachi K. (2003). Preventing musculoskeletal disorders in clinical dentistry: Strategies to address the mechanisms leading to musculoskeletal disorders. J. Am. Dent. Assoc..

[69] Pirvu C., Patrascu I., Pirvu D., Ionescu C. (2014). The dentist’s operating posture—Ergonomic aspects. J. Med. Life.

